# The Reception of the Armistice

**Published:** 1918-11-16

**Authors:** 


### THE WORST WAR OF THE WORLD.

### XI. THE RECEPTION OF THE ARMISTICE.

It is a remarkable fact that in this, the worst war of the world, the fighting on all Fronts should have ceased at 11 a.m. on November 11 by the signing of an armistice at 5 a.m. after a discussion which was prolonged all night. It is notable, as *The Times* correspondent records, that at Sedan when "Cease Fire" sounded the usual tales of German brutality, especially to British prisoners, were testified to by Frenchmen (ordinary men of middle-age) whose voices broke and tears flowed while they tried to tell of things they had seen, and to give proper voice to their pity for our men. At Sedan all through the night and up to the moment when the armistice began the artillery and machine-guns on both sides were busily at work as though there was no thought of hostilities coming to an end. Until past one o'clock in the day there was a thick white mist over the whole district, which hid everything for a distance of twenty yards from the observer. Here, on the cessation of hostilities, there was an uncanny silence. Everywhere the paraphernalia of war surrounded the observer, and the air was absolutely still, the silence being unbroken by a single shot. Small wonder that the fighting men themselves should exhibit the effects of the four years' struggle when the four years' noise came to an end. It is recorded that with the troops at Sedan there was nothing to do except to be glad; and they were glad. This was written on the face of every one of the thousands of men coming and going on the crowded roads leading to the town. For although the armistice had come the huge war machine worked steadily and regularly on all day long. Infantry, Artillery, and Engineers were coming away from the Front, and reliefs going back. It was a marvellous sight, *The Times* records, and a wonderful memory, but oh, how glad were the men to whom, in God's mercy, we owe victory and triumph!

In the Metropolis of the Empire there was the same feeling. Joy everywhere; hearts full of gratitude, to which, as Mr. Lloyd George declared, no tongue could give adequate expression. All the morning the King and Queen had enthusiastic greetings from the great crowds which gathered outside Buckingham Palace, and in the afternoon the people assembled again, sang patriotic songs, and shouted in unison "We want King George." At half-past three the King and Queen drove out in an open carriage, accompanied by Princess Mary, and though the rain was falling it did not matter. The carriage was driven at a slow pace, and the route taken was by The Mall, through the Strand, Fleet Street, Ludgate Hill, and Queen Victoria Street, to the Mansion House, and thence back to the Palace by Holborn, along Oxford Street, Shaftesbury Avenue, through Trafalgar Square, and under the Admiralty Arch to Buckingham Palace. Everywhere Their Majesties were greeted by shouts of welcome, and the demonstration throughout was of the heartiest possible description.

The House of Commons assembled at the usual hour, and amongst the important business to be done was a vote of credit for hundreds of millions sterling. But this and all other business was placed on one side, and immediately after prayers the Prime Minister entered the House, which was packed, there being a large attendance of peers; the only deserted portion, by a strange coincidence, being the Strangers' Gallery, the people throughout the land being absorbed with the joy of thanksgiving and thankfulness. Cheers came everywhere from the hearts of the people as from those assembled in the House of Commons, and it is worthy of permanent record that, the clause in the armistice which drew the deepest and most sustained cheer, was that which provided for the immediate repatriation of all Allied prisoners of war. Mr. Lloyd George was greeted, at the proudest moment of his career, with cheering of a kind rarely heard at St. Stephen's. Mr. Asquith and the ex-Ministers who sat with him joined heartily in this spontaneous outburst, and the colleagues of the Prime Minister acclaimed their chief with all the fervour of personal affection. Then Mr. Lloyd George, being once more heartily cheered, rose to read the terms of the armistice, which were received with intense satisfaction, for it was felt that the Allies had achieved security. Mr. Lloyd George's voice was broken with emotion when he said:—

"This is no time for words. Our hearts are too full of gratitude, to which no tongue can give adequate expression." Thus has come to an end, the Prime Minister impressively declared, "the cruellest and most terrible war that has ever scourged mankind." His devout hope was that all wars were ended, a sentiment upheld by Mr. Asquith with a full heart, for the armistice had made it impossible for the war to be resumed. Then the House gratefully accepted the Prime Minister's suggestion, that all should at once proceed to St. Margaret's Church to give thanks for the deliverance of the world from its great peril. A procession was formed, with the Speaker at its head, the Prime Minister and Mr. Asquith immediately following, who were accompanied by the Commons. They filed out through St. Stephen's Hall across the road, which was densely crowded, into the church. Then came the House of Lords in procession, led by the Lord Chancellor.

No one who was present at the service at St. Margaret's is ever likely to forget the occasion. Without any formal order of service, without any sermon, those present gave themselves up to an hour of prayer and thanksgiving, of humble supplication and radiant gratitude. Commencing with the Hundredth Psalm, the Commons Chaplain, Canon Carnegie, read beautiful prayers of thankfulness for victory and for the comfort of the bereaved. Then was sung "O God, our help in ages past," followed by the reading of the

☛ Previous articles appeared on Feb. 9, 23, March 9, 23, April 6, 20, May 4, 25, June 8, 22, and Aug. 10, pp. 412-13.

**The Worst War of the World**—*(continued)*

lesson from the sixty-first chapter of Isaiah, with the two notable verses, "He hath sent me to bind up the broken-hearted, to proclaim liberty to the captives, and the opening of the prison to them that are bound. They shall raise up the former desolations, and they shall repair the waste cities." Then came the glad strains of the *Te Deum*, followed by the Primate, who gave the Benediction. The service concluded with the National Anthem, sung, as it ought always to be sung, with heart and soul by everyone present. As the Lords and Commons left the church they passed the simple Roll of Honour which the Commons had placed in the church to the memory of their heroic dead. So, as *The Times* records, "their last thoughts on this memorable day were as their first, the saddest of all memories merging into the glory of the battle won."

The reception of Their Majesties, the proceedings in the House of Commons, and the service in St. Margaret's Church, attended by the Lords and Commons, show that the keynote of the feeling of the people throughout the Metropolis of the country was typical of that of the whole Empire, and of the people of the United States and of our Allies everywhere. The spirit exhibited was in no sense a note of exaltation, but the joy of thanksgiving, and with it an overmastering sense of compassion which made the proceedings, in reality, a solemn act of consecration. There is here vital evidence that victory has created an overwhelming resolution and desire, that the termination of the war may be followed by a universal peace, never, it is hoped, to be broken or disturbed so long as the world endures.

If ever there was a doubt in any intelligent person's mind that this war has been God's War, especially the happenings of the last six months should remove that impression, and bring the whole of the people of the world to recognise Almighty God, as the Father of all, Who rules and governs with wisdom, is full of mercy and compassion, and sees and knows all things.

